# Magnetic resonance imaging in size assessment of invasive breast carcinoma with an extensive intraductal component

**DOI:** 10.1186/1471-2342-9-5

**Published:** 2009-04-07

**Authors:** Arjan P Schouten van der Velden, Carla Boetes, Peter Bult, Theo Wobbes

**Affiliations:** 1 Department of Surgical Oncology, Radboud University Nijmegen Medical Centre, Nijmegen, the Netherlands; 2 Department of Radiology, Maastricht University Medical Centre, Nijmegen, the Netherlands; 3 Department of Pathology, Radboud University Nijmegen Medical Centre, Nijmegen, the Netherlands

## Abstract

**Background:**

Breast-conserving treatment of invasive breast carcinoma with an extensive intraductal component (EIC) is associated with DCIS-involved surgical margins and therefore it has an increased recurrence rate. EIC is a non-palpable lesion of which the size is frequently underestimated on mammography. This study was undertaken to evaluate the accuracy of MRI in size assessment of breast cancer with EIC.

**Methods:**

23 patients were identified and the mammographic (n = 21) and MR (n = 23) images were re-reviewed by a senior radiologist. Size on MR images was compared with histopathological tumour extent.

**Results:**

The correlation of radiological size with histopathological size was r = 0.20 in mammography (p = 0.39) compared to r = 0.65 in MRI (p < 0.01). Mammography underestimated histopathological tumour size in 62%. MR images over- or underestimated tumour size in 22% and 30% of the cases, respectively. In poorly differentiated EIC, MRI adequately estimated the extent more often compared to moderately differentiated EIC (60% versus 25%, respectively).

**Conclusion:**

Size assessment of MRI imaging was more accurate compared to mammography. This was predominantly true for poorly differentiated EIC.

## Background

Breast-conserving surgery has become a standard therapy for early-staged, resectable breast carcinomas [1]. An important factor in achieving local tumour control and patient survival is the adequacy of the excision. Patients with tumour-involved surgical margins have lower overall survival rates compared to patients with completely excised breast cancers [2-4].

It has been shown that breast-conserving treatment of breast cancer with an extensive intraductal component (EIC) gives rise to a higher recurrence rate which is most likely due to the presence of residual ductal carcinoma in situ (DCIS) [5-7]. Holland and colleagues have shown that primary breast tumours with EIC were more likely to have residual DCIS anywhere in the remaining breast compared to invasive breast cancers without an EIC [8].

EIC is found in 30%–40% of all invasive breast carcinomas [7-9]. Additionally, EIC is non-palpable which makes a microscopically complete resection technically difficult.

This makes an accurate preoperative measurement of invasive breast cancer with surrounding DCIS mandatory in order to decrease the need of secondary interventions caused by tumour-involved surgical margins. Even though DCIS is visible on a mammography, its histological size is frequently underestimated [8,9].

For invasive breast carcinoma, magnetic resonance imaging (MRI) has been shown to accurately predict tumour size and multifocality with high sensitivity [10-15].

The current study was undertaken to evaluate whether the size of breast carcinomas with EIC can be accurately measured on MRI images.

## Methods

We selected 23 female patients from the database of the Pathology department with a histopathologically confirmed diagnosis of an invasive breast carcinoma and a DCIS component who underwent a MRI breast examination in the period between January 2000 and December 2004. During this period, MRI was randomly performed and no specific criteria were used whether patients underwent a MRI or not. During the study period, performance of MR breast imaging was not standard care. Currently though, MRI is routinely used as standard work-up for breast malignancies in our institute.

As in this retrospective study patient care has been evaluated, no ethical approval was necessary according to our local policy. Similarly, no informed consent was obtained.

Patients with DCIS and a micro-invasive carcinoma (size of invasive carcinoma of 1 mm or less) were also considered eligible.

Tumours were considered EIC positive when DCIS was predominant lesion and when DCIS was clearly extending beyond the infiltrating carcinoma, according to the definition of Holland and colleagues [8].

The medical records, mammographic and MR images of all 23 patients were retrospectively reviewed and all available clinical, radiological, and pathological data were collected. Items noted were tumour diagnosis, location of the lesion, preoperative histopathological diagnosis, number of surgical procedures, definitive surgical treatment, histopathological grading of in situ and invasive carcinoma, and the percentage of EIC of the entire lesion.

A senior radiologist (C.B.) experienced in reading breast images (approximately 1500 MR breast images per year) reviewed both mammography and MRI images without knowledge of the histopathological findings.

Mammographic examination (Senographe 2000, GE Medical systems) constituted of standard oblique and craniocaudal projections. Images were reviewed for presence of abnormalities by microcalcifications, masses, or architectural distortions.

MR images were made at field strength of 1.5 Tesla (Symphony, Siemens) with the patient placed in a prone position with the breasts hanging in a double-breast coil.

The scanning protocol consisted of 1 pre-contrast FLASH 3D acquisitions at a high spatial resolution (TR/TE 7.8/4, FA 20, rectangular FOV 340, matrix 256*256, slice thickness 1.3, orientation coronal, AT 90s). Thereafter, the FLASH 3D sequence was repeated 5 times after intravenous administration of contrast agent (0.2 mmol gadolinium per kilogram of body weight). Subsequently subtraction images were created from the pre-contrast and the second post-contrast acquisition.

The subtracted FLASH 3D images were viewed on a Dynacad working station (InVivo) and used for evaluation of both morphological and dynamic imaging parameters. Morphological pattern of lesions was classified as round or oval, irregular or ductal whereas enhancement was noted as heterogeneous or homogeneous. Margins were categorised as regular, irregular or spiculated. Kinetic contrast-enhancement characteristics over time were classified as progressive, plateau, or washout. Criteria for suspicious enhancement during time were defined as a signal increase followed by a plateau or washout phase.

The results of both mammography and MRI were scored according to the Breast Imaging Reporting and Data System (BI-RADS) classification [16]. Categories were: category 1, negative; category 2, benign finding; category 3, probably benign finding, follow-up requested; category 4, suspicious abnormality, and category 5, highly suggestive of malignancy.

Histopathological examination and sampling of the excisional biopsy specimens were based on specimen X-rays of the whole and sliced specimens. Handling of the mastectomy specimens was based on the correlated radiographic and pathologic technique developed by Egan, which has been routinely performed in our pathology department for many years [17]. This method is described in detail elsewhere [18]. The specimens were cooled and sliced in serial sections with approximately 5-mm intervals. Radiographs were made from the tissue slices. Suspicious lesions and randomly selected areas from each quadrant and the nipple were sampled.

The histopathological extent of the tumour was measured as the total diameter of the intraductal component in which the invasive carcinomas were located. For those patients who underwent a re-excision, the extent of the tumour within the re-excised specimen was recorded and added to the tumour size of the first excision. The total tumour size therefore included both the in situ and the invasive component.

Lesion sizes as measured by the different methods were categorized in groups by 5 mm (0–5 mm, 6–10 mm, etc). A difference of 10 mm or less between size assessed on imaging and size at histopathological examination is considered as an adequate measurement. The Spearman correlation coefficient was calculated to analyse the size assessment at mammography, MRI, and histopathological examination.

The data were analysed with SPSS version 11.5 (SPSS, Chicago, IL, USA). For all statistical analyses a p-value of < 0.05 was considered statistically significant.

## Results

Table 1 lists patient and tumor characteristics of the study population. The median age at diagnosis was 51 years (range: 28–68 years). Final treatment constituted of a mastectomy in the majority of patients (91%). After definitive treatment, surgical margins were free of tumour in 22 patients whereas in one patient the surgical margin was focally involved.

**Table 1:** Patient and tumor characteristics of the study population (n = 23).

Characteristic	Category	N	%
Tumour diagnosis	Clinical symptoms	5	22
	Mammography	16	70
	MRI	2	8
Location of the tumour	Inner quadrant	2	9
	Outer quadrant	17	74
	Central	4	17
Preoperative diagnosis of histological core needle biopsy	DCIS	5	21
	DCIS with invasive carcinoma	14	61
	Not conclusive	2	9
	Not performed	2	9
Number of surgical procedures	1	17	74
	2	5	22
	3	1	4
Final treatment	Breast-conserving surgery	2	9
	Mastectomy	21	91
Axillairy staging	Sentinel lymph node biopsy	10	44
	Axillairy lymph node dissection	10	44
	Sentinel lymph node biopsy followed by axillairy		
	lymph node dissection	3	13
Grading of DCIS	Grade I, well differentiated	0	0
	Grade II, moderately differentiated	8	35
	Grade III, poorly differentiated	15	65
Grading of invasive carcinoma	Grade I, well differentiated	8	35
	Grade II, moderately differentiated	6	26
	Grade III, poorly differentiated	9	39
Size of invasive carcinoma	≤ 1 mm	4	17
	1–10 mm	8	35
	11–20 mm	4	17
	21–30 mm	5	22
	31–40 mm	1	4
	41–50 mm	1	4
Percentage EIC	<50%	5	22
	50%–75%	6	26
	>75%	12	52

DCIS: ductal carcinoma in situ; EIC extensive intraductal component

Preoperative mammographic examination was performed in all but two patients whereas all 23 patients underwent MR breast imaging.

Mammographic images revealed abnormalities (defined as a BI-RADS classification 4 or 5) in 17/21 examinations (81%). On two mammograms (10%), no abnormalities were seen and findings on mammographic exams of another two patients were classified as 'probably benign' (one patient with microcalcifications and one patient with a density). These last four exams (19%) are thus considered as false-negative findings. The predominant mammographic findings were microcalcifications with or without a density or an architectural distortion (62%).

**Figure 1:**
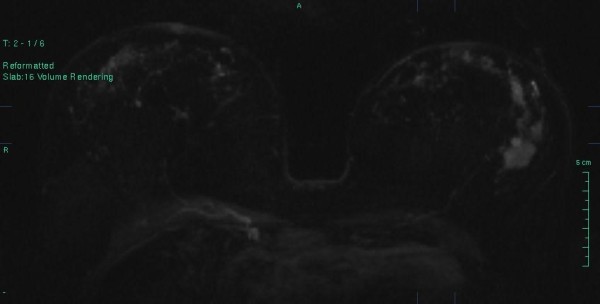
**MR image demonstrating an inhomogeneous spiculated mass of 30 mm within the left breast**. At histopathological examination a 85 mm moderately differentiated invasive ductal carcinoma with surrounding moderately differentiated DCIS was found.

The specific findings on MRI are listed in detail in table 2. An example of a MR image is presented in figure 1.

**Table 2:** MRI findings of the study population (n = 23).

Characteristic	Category	N	%
Morphologic pattern	Round or oval	2	9
	Lobulated	2	9
	Irregular	4	17
	Ductal	7	30
	Segmental	8	35
Enhancement	Heterogeneous	23	100
	Homogeneous	0	0
Margins	Regular	3	13
	Irregular	11	48
	Spiculated	9	39
Kinetic contrast enhancement	Progressive	7	30
	Plateau	1	4
	Washout	13	57
	Unknown^1^	2	9
BI-RADS classification	4: suspicious abnormality	9	39
	5: highly suggestive of malignancy	14	61

^1 ^MR images of 2 patients were reviewed on a plain film MRI on which no kinetic contrast enhancement could be assessed.

In two patients, MR images were available on plain films only. In these patients, therefore, no data on contrast enhancement kinetics are presented. All MR exams showed abnormalities which after reviewing were classified as either 'suspicious' or 'highly suggestive of malignancy' according to the BI-RADS classification (score 4 and 5, respectively). Margins of the lesions on MRI were irregular or spiculated in the majority of tumours (87%).

Histopathological examination of the excised breast specimen revealed both invasive and in situ breast carcinoma in all patients. The majority of tumours were composed of an area with in situ carcinoma in which an invasive carcinoma was located. In four tumours (17%), the size of the invasive carcinoma was 1 mm or less and these lesions were considered as micro-invasive carcinomas.

In two cases, the invasive tumour and DCIS were adjacently located and for these lesions, the total tumour size was calculated as the sum of DCIS size and invasive tumour size. In another three patients, the tumour size was calculated by adding the size of re-excised residual tumour to the size of the initial excised lesion.

Mean whole tumour size for the study population was 49 mm (median 45 mm, range: 18–105 mm). The EIC accounted for 50% or more of the complete tumour size in 18 patients (table 1).

**Figure 2:**
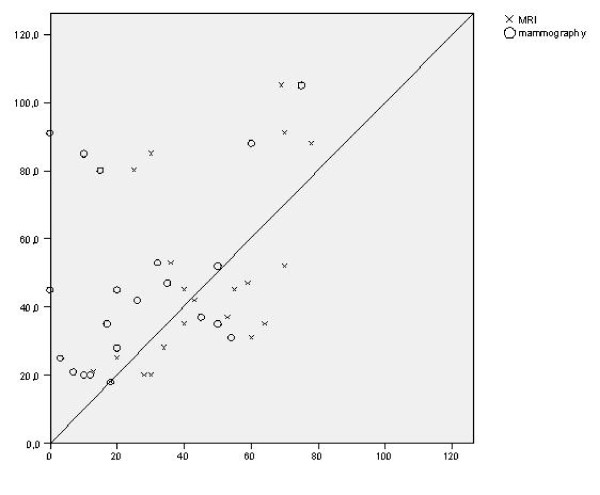
Scatter diagram and the correlation of diameter on radiology (x-axis) with histopathological size assessment (y-axis)

Table 3 and figure 2 present the results of size assessment by mammography and MRI compared to the histopathological size measurements. These results are expressed as underestimation, adequate measurement, or overestimation of the histopathological size.

**Table 3:** Size assessment of invasive breast carcinoma with an extensive DCIS component by mammography (n = 21) and MRI (n = 23) compared to histopathological size.

Radiological size assessment		Mammography N (%)	MRI N (%)
Underestimation	> 50 mm	4 (19)	3 (13)
	30–49 mm	1 (5)	1 (4)
	20–29 mm	4 (19)	1 (4)
	11–19 mm	4 (19)	2 (9)
Adequate measurement*		6 (28)	11 (48)
Overestimation	11–20 mm	1 (5)	3 (13)
	21–30 mm	1 (5)	2 (9)

* A difference of 10 mm or less between size assessed on imaging and size at histopathological examination is considered as an adequate measurement.

An adequate measurement, defined as a difference between histopathological size and radiological size of 10 mm or less, was found in six mammographic exams (28%) compared to 11 MRI exams (48%). The correlation of mammographic tumour size with histopathological size was r = 0.20 (p = 0.39). For MRI size assessment, the correlation with histopathological tumour size was r = 0.65 (p < 0.01).

Mammography underestimated histopathological tumour size in the majority of cases (13, 62%). In contrast, MR imaging was equally like to over- or underestimate estimate tumour size: in five and seven patients, respectively. Of tumours surrounded by poorly differentiated DCIS, MRI size assessment was adequate in 60% (9/15) compared to 25% (2/8) in moderately differentiated DCIS. In the moderately differentiated EIC, MRI underestimated tumour size in half of cases (4/8) compared to 20% (3/15) in poorly differentiated EIC (p = 0.23).

## Discussion

As breast conservation and prevention of recurrent disease are the main goals in treatment of early resectable breast cancer and as presence of an EIC is a risk factor for recurrent disease, it is important to reliably identify the tumour extent preoperatively.

This retrospective analysis on size assessment of invasive carcinoma with EIC revealed that this type of breast tumours can be visualised on MRI and we found a significant correlation between tumour extent on MRI and the size measured at histopathological examination. Overall, size assessment on MRI was more accurately when compared to mammography. Mammographic determination of the extent of DCIS and, therefore, EIC mainly depends on the presence of microcalcifications [9,19,20] However, mammographic estimates, based on the extent of microcalcifications, frequently underestimates tumour size [8,9] Additionally, mammography does not reliably demonstrate the extent of uncalcified DCIS which is supported by the findings of the current analysis.

Enhancement of malignant tumours on MRI is caused by the presence of tumor-induced angiogenesis. An increased density of microvessels will increase blood flow, which causes contrast enhancement. Furthermore, tumor-induced microvessels demonstrate structural abnormalities which give rise to leakage of contrast medium. This causes the characteristic malignant contrast-enhancement kinetics (plateau and washout phenomenon) [21-23].

An increased amount of stromal microvessels has been shown for DCIS [21]. Gilles et al. showed contrast enhancement in DCIS lesions and micro-invasive carcinomas and subsequently demonstrated tumor angiogenesis in enhancing lesions [23].

This makes MR breast imaging able to detect both calcified and uncalcified DCIS [15,24,25].

And, therefore, invasive breast cancer with EIC can be visualised on MR breast imaging. Reported detection rates of MRI for invasive cancer with EIC differs between 45%–100% (table 4) [12,25-34].

**Table 4:** Detection rate of MRI for invasive breast carcinoma and an extensive intraductal component (EIC).

First author	Histopathology	MRI detection rate (%)
Mumtaz^12^	N = 19	14/19 (74%)
Soderstrom^25^	N = 11	11/11 (100%)
Kerslake^27^	N = 13	7/13 (54%)
Satake^28^	N = 15	14/15 (91%)
Hata^29^	N = 68	54/68 (79%)
Komatsu^30^	N = 19	16/19 (84%)
Ikeda^31^	N = 59	42/59 (71%)
Sundarajan^32^	N = 28	14/28 (50%)
Shimauchi^33^	N = 44	33/44 (75%)
Sundarajan^34^	N = 20	9/20 (45%)
VanGoethem^35^	N = 50	34/50 (68%)

EIC extensive intraductal component

The morphologic pattern of lesions on MRI were ductal or segmental in 15/23 (65%) lesions, whereas margin enhancement on MRI was irregular of spiculated in 20/23 (87%) tumours. This probably reflects the extension of DCIS and has been reported by others as well [12,25,29,30,34].

Data on size assessment of breast carcinomas with EIC are scarce. Correlation coefficients between MRI tumour size and histopathological extent reported differ between r = 0.42 and r = 0.87 [28,30,34]. This is in concordance with results of the presented study (r = 0.65).

In the presented study population however, the size of EIC was underestimated on MRI in 30% (7/23). There also was a trend towards more underestimation of moderately differentiated EIC compared to poorly differentiated EIC on MRI, though this reached no statistical significance. This has also been found by others: in the series of Goethem et al. five out of 12 low grade EIC (42%) were not depicted in MRI compared to 11 out of 38 high grade EIC tumours (28%) [34].

These false-negative findings could be explained by microvessel density: in the previously mentioned study of Gilles and colleagues the two false-negative cases were found to exhibit weak tumor angiogenesis in the stroma around the ducts involved by DCIS [23]. Similarly, it has been shown that high grade DCIS (i.e. poorly differentiated tumours) has an higher microvessel density when compared to non-high grade lesions [21,22].

Size overestimation of DCIS and EIC by MRI has been described previously [15,24,28,34,35]. In the presented population, MRI overestimated approximately 20 per cent of EIC tumours. Overestimation of tumour extent is in the majority of cases due to false-positive enhancement of benign proliferative processes such as fibrocystic changes or adenosis [12,15,23,25,35].

The most important clinical issue however is whether MRI has in beneficial effect on outcome of treatment. In the study of Berg and colleagues, only MRI depicted DCIS in 6 out of 19 breasts with EIC positive carcinomas, whereas in five of these breast conserving surgery was initially anticipated [15].

Recently, the relation between preoperative MRI and outcome after breast-conserving treatment for early stage (T1 or T2) and in situ breast carcinoma was determined in a large non-randomised cohort study [36]. It was concluded that the use of MRI was not associated with an improved outcome after breast-conserving treatment.

## Conclusion

This report shows that invasive breast cancer with EIC can be visualised on MRI. In contrast to mammography, tumour extent measured on MRI correlates significantly with histopathological tumour size, but, tumour size is underestimated frequently, which was most obviously seen in poorly differentiated EIC. This study has some limitations to be addressed. It is a retrospective review of non-consecutive patients with a proven diagnosis of an EIC breast carcinoma. The number of patients is relatively small, which makes firm statistical conclusions difficult.

Therefore, future research is mandatory to explore the value of MRI in breast cancer with EIC. The most important issue to address is whether MRI is able to improve outcome by decreasing the need for re-operations and lowering recurrence rates.

## Competing interests

The authors declare that they have no competing interests.

## Authors' contributions

ASvdV collected the data, reviewed the images and drafted the manuscript. CB collected the data and reviewed the images. PB collected data and helped drafted the manuscript. TW designed the study and helped drafted the manuscript. All authors approved the manuscript.

## Pre-publication history

The pre-publication history for this paper can be accessed here:

http://www.biomedcentral.com/1471-2342/9/5/prepub
